# Synaptic Responses Evoked by Tactile Stimuli in Purkinje Cells in Mouse Cerebellar Cortex Crus II *In Vivo*

**DOI:** 10.1371/journal.pone.0022752

**Published:** 2011-07-26

**Authors:** Chun-Ping Chu, Yan-Hua Bing, Quan-Ri Liu, De-Lai Qiu

**Affiliations:** 1 Cellular Function Research Center, Yanbian University, Yanji, Jilin Province, China; 2 Department of Physiology and Pathophysiology, College of Basic Medicine, Yanbian University, Yanji, Jilin Province, China; 3 College of Basic Medicine, Function Experiment Center, Yanbian University, Yanji, Jilin Province, China; Tokyo Medical and Dental University, Japan

## Abstract

**Background:**

Sensory stimuli evoke responses in cerebellar Purkinje cells (PCs) via the mossy fiber-granule cell pathway. However, the properties of synaptic responses evoked by tactile stimulation in cerebellar PCs are unknown. The present study investigated the synaptic responses of PCs in response to an air-puff stimulation on the ipsilateral whisker pad in urethane-anesthetized mice.

**Methods and Main Results:**

Thirty-three PCs were recorded from 48 urethane-anesthetized adult (6–8-week-old) HA/ICR mice by somatic or dendritic patch-clamp recording and pharmacological methods. Tactile stimulation to the ipsilateral whisker pad was delivered by an air-puff through a 12-gauge stainless steel tube connected with a pressurized injection system. Under current-clamp conditions (I = 0), the air-puff stimulation evoked strong inhibitory postsynaptic potentials (IPSPs) in the somata of PCs. Application of SR95531, a specific GABA_A_ receptor antagonist, blocked IPSPs and revealed stimulation-evoked simple spike firing. Under voltage-clamp conditions, tactile stimulation evoked a sequence of transient inward currents followed by strong outward currents in the somata and dendrites in PCs. Application of SR95531 blocked outward currents and revealed excitatory postsynaptic currents (EPSCs) in somata and a temporal summation of parallel fiber EPSCs in PC dendrites. We also demonstrated that PCs respond to both the onset and offset of the air-puff stimulation.

**Conclusions:**

These findings indicated that tactile stimulation induced asynchronous parallel fiber excitatory inputs onto the dendrites of PCs, and failed to evoke strong EPSCs and spike firing in PCs, but induced the rapid activation of strong GABA_A_ receptor-mediated inhibitory postsynaptic currents in the somata and dendrites of PCs in the cerebellar cortex Crus II in urethane-anesthetized mice.

## Introduction

The cerebellar cortex receives a wide variety of sensory inputs and generates motor-related outputs. To understand the integration of sensory and motor information performed by the cerebellar cortex, it is essential to determine the synaptic properties of Purkinje cells (PCs) in response to tactile stimuli.

The cerebellum receives two distinctly different types of excitatory afferents: the mossy fibers and the climbing fibers. Both types of fibers transfer sensory information to the cerebellar cortex. Activation of climbing fibers evokes complex spike firing of the PCs, whereas activation of the mossy fibers evokes simple spike firing of PCs via the parallel fibers [1]–[6]. The cerebellar PC is the computation center, receiving convergent projections from all other cortical neurons and providing the sole output from the cerebellar cortex [5]. The classical view of the functional organization of the cerebellar cortex asserted that the sensory information coming from mossy fibers induces the beam-like excitation of parallel fibers (PFs) and their innervated PCs [7], [8]. Activation of PCs underneath the PF beam has been induced with direct electrical stimulation of the molecular layer [9]–[11], but more natural stimulation of the mossy fiber inputs has failed to activate beams of PCs [2], [12]–[14]. A modeling study supported by *in vivo* experiments has shown that tactile stimulation-induced beams of PCs require a blockade of GABA_A_ receptor-mediated inhibition. These d–ata suggest that inhibition plays a complex and central role in the physiological and functional organization of the cerebellar cortex [14], [15].

The structure of the inhibitory interneuron network in the mammalian cerebellar cortex has been previously described in detail [5], [1]–[20]. Basket and stellate cells are activated by the PFs [21]–[23]. Inhibitory axons of stellate cells selectively innervate the dendrites of PCs, whereas the inhibitory axons of basket cells project to the initial segment of the axon to form pinceau synapses and initiate perisomatic inhibition of PCs [5], [24]. The molecular layer interneurons are small and have high-input resistance, which leads to a low threshold for activation [25].

We previously reported that an air-puff stimulation on the ipsilateral whisker pad failed to evoke complex spikes and simple spike firing, but induced a GABA_A_ receptor-mediated pause in spike firing in PCs in the cerebellar cortex folium Crus II [26]. To explore the properties and to characterize the synaptic responses of the cerebellar PCs in response to sensory stimulation, we here used somatic and dendritic patch-clamp recording techniques and pharmacological methods to study the synaptic currents in cerebellar PCs in response to tactile stimulation in urethane-anesthetized mice. We found that air-puff stimulation on the ipsilateral whisker pad induced asynchronized PF excitatory inputs onto dendrites of PCs. Air-puff stimulation failed to provoke spike firing in PCs, but induced GABA_A_ receptor-mediated inhibitory postsynaptic potentials (currents) in the somata and dendrites of PCs. We propose that this inhibition plays a crucial role in controlling the output of sensory information in PCs in the mouse cerebellar cortex Crus II.

## Results

### Effects of tactile stimulation on PC membrane potential and currents

**Figure 1:**
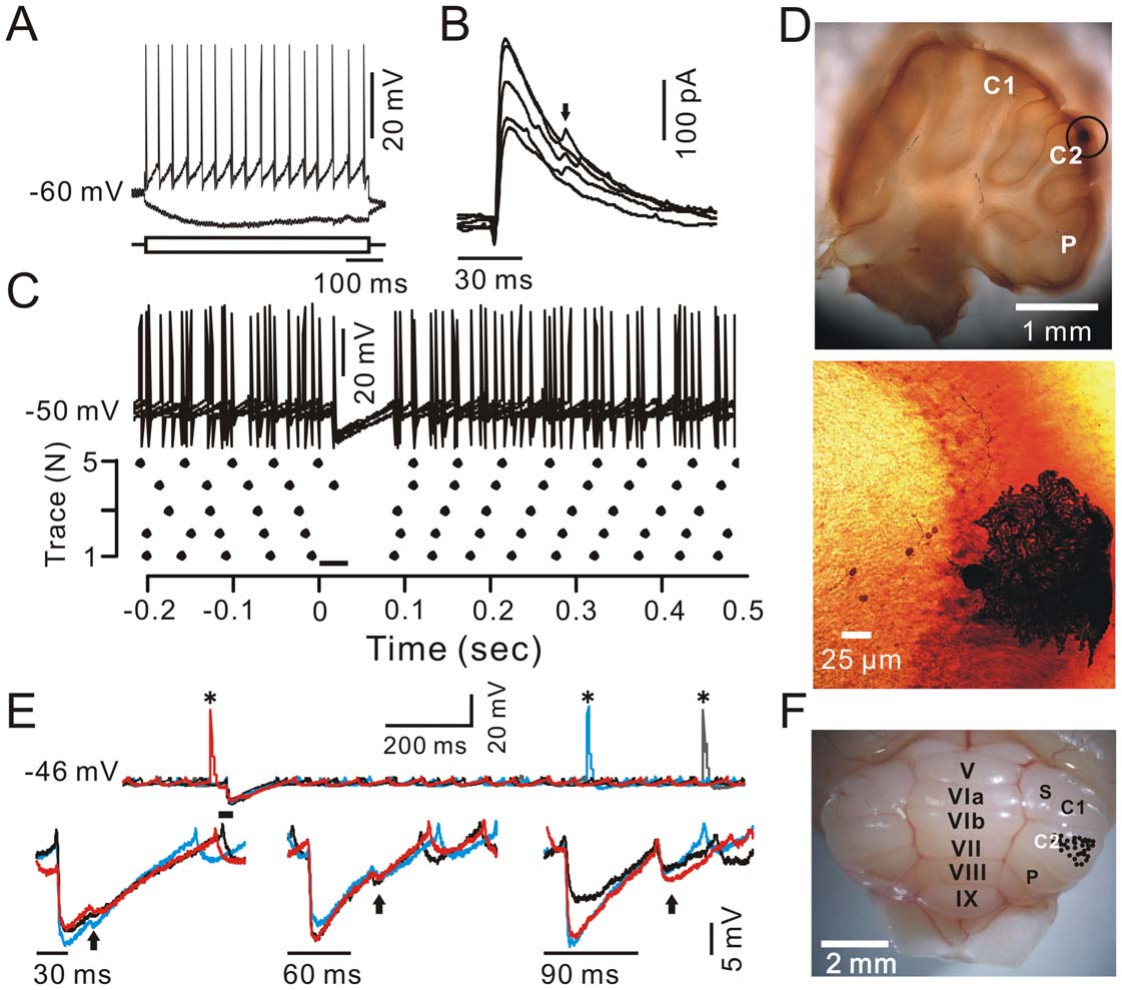
PC somata and dendrites in response to tactile stimulation. A, Whole-cell patch-clamp recording from a soma of a PC in response to hyperpolarizing (–100 µA) and depolarizing (+100 µA) current pulses. B, Under voltage-clamp (V_hold_  =  –70 mV), superposition of five sweeps shows the effects of tactile stimuli (bar, 30 ms) on PC membrane currents. Notably, 3/5 IPSC traces expressed a second peak (arrow) at ∼30 ms after the first peak. C, Under current-clamp conditions (I = 0), superposition of five sweeps (upper) and the raster plot of simple spike events (lower) show how the PC responds to tactile stimuli (bar, 30 ms). D, The photomicrographs show the morphology of the cell, which is shown in A–C. The left column shows an overview of the location of the biocytin-labeled cell, which is indicated with a black circle. The right column shows the detail of the biocytin-labeled cell. E, Under current-clamp (I = 0), a representative dendritic patch-clamp recording shows the response of the PC dendrite to tactile stimuli. Upper, superposition of five sweeps presenting the dendrite in response to tactile stimuli (bar, 30 ms). Asterisks indicate complex spikes. Lower, superposition of three sweeps showing the different durations (30 ms, 60 ms, and 90 ms) of tactile stimulation-induced responses in the dendrites. Arrows indicate the offset responses. F, *Ex vivo* image of cerebellum with lobules indicated by letters, C_1_ is Crus I, C_2_ is Crus II; P, paramedian lobules; S, simple lobule. The overall distribution of the recorded PCs in this study is indicated by black circles; it is located in Crus II.

Thirty-three PCs were analyzed under whole-cell patch-clamp recording configurations (Fig. 1A), and confirmed by biocytin histochemistry (Fig. 1D). Thirty-one PCs (93.9%) expressed responses to the air-puff stimulation with a mean latency of 17.0±0.2 ms, and exhibited a strong inhibition rather than an excitation. Under voltage-clamp conditions (V_hold_  =  –70 mV; Fig. 1B), tactile stimuli evoked a sequence of transient inward currents (11.8±3.3 pA; n = 12 cells) followed by strong outward currents (127.5±20.2 pA; n = 12 cells), which were identified as inhibitory postsynaptic currents (IPSCs). The time constant of decay for the IPSCs was 43.7±4.8 ms, and the 10–90% rise time was 1.6±0.1 ms (n = 12 cells). The outward currents were not reversed at the holding potential of −70 mV, suggesting poor space clamping of PCs under *in vivo* conditions. Although the somatic membrane potential may be well controlled, the membrane potential of the axon initial segment and dendrites may be poorly controlled. In addition, some current traces (26/55 traces) expressed a second peak of outward currents (Fig. 1B, arrow) with a mean delay of 28.7±0.6 ms (n = 11 cells) from the first peak. This mean delay was similar to the delay of the air-puff (30 ms), which suggests that the second responses were induced by stimulation offset. Under current-clamp conditions (I = 0), air-puff stimuli evoked neither simple spikes nor complex spikes, but induced strong inhibitory postsynaptic potentials (IPSPs) in the somata of PCs (Fig. 1C) with a mean IPSP amplitude of 9.7±1.0 mV (n = 12 cells). Notably, the tactile stimulation also evoked strong IPSPs (6.0±1.2 mV; n = 19) in the dendrites of PCs without effects on complex spike activities (Fig. 1E, upper). The dendritic IPSPs also expressed the second peak with a mean delay of 28.7±0.5 ms, 58.6±0.7 ms, and 89.0±1.0 ms (n = 3 cells) from the first peak. These data correspond to the durations of the air-puff of 30 ms, 60 ms and 90 ms, respectively (Fig. 1E, lower) and confirmed that the second peak was evoked by stimulation offset. The probability of the offset responses increased when the stimulation duration was extended; 93% (28/30 traces; n = 3 cells) of recordings expressed the offset responses when the stimulation duration was up to 90 ms. Thus, the tactile stimulation evokes inhibition rather than excitation in the somata and dendrites of PCs. This inhibition is induced by stimulation onset and is evoked also by stimulation offset. The overall distribution of the recorded PCs in this study is shown in Figure 1F; the location of the recorded PCs was the mouse cerebellar cortex Crus II, as in our previous study [26].

### Pharmacological properties of the somata of PCs in response to tactile stimulation

**Figure 2:**
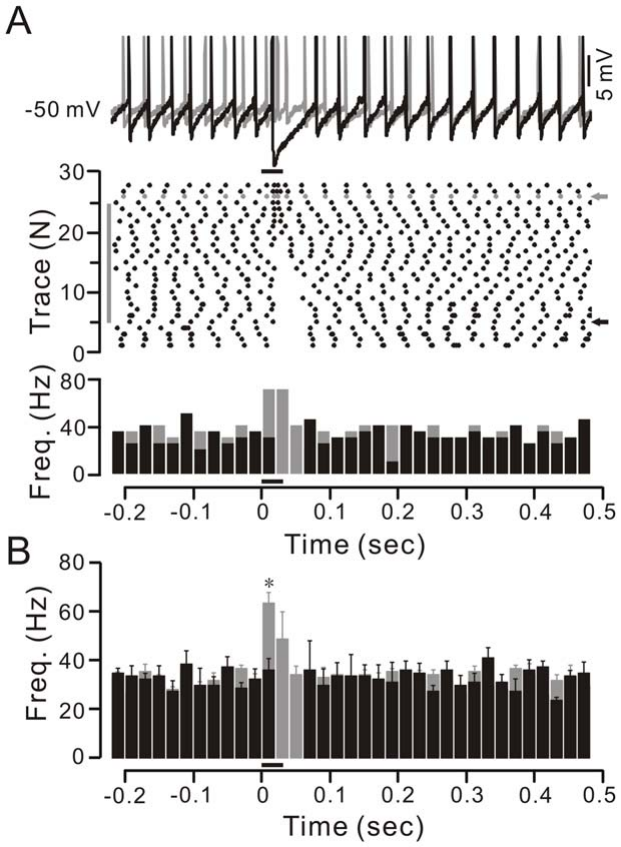
Response properties of the somata of PCs during tactile stimulation under current-clamp. A, Representative examples (upper; arrows shown in middle), raster plot (middle) and instant frequency (lower) of simple spike events showing a PC response to tactile stimulation (black bar, 30 ms) before (black) and after application of 20 µM SR95531 (gray). The gray bar indicates the application of SR95531 (10 min). B, Pooled data (n = 6) indicate the time course of PC simple spike firing rate in response to tactile stimulation (black bar, 30 ms) in ACSF (black) and with SR95531 (gray). Note that the frequency of the simple spike firing was significant increased after stimulus, which was significantly higher than the basal simple spike firing rate (asterisk; p = 0.005; n = 6). Error bars indicate ± SEM.

Under current-clamp conditions (I = 0), tactile stimuli evoked strong IPSPs (Fig. 2A) with a pause of simple spike firing at a mean value of 75.8±6.2 ms (n = 12 cells), which was similar to cell-attached recordings (76.2±6.5 ms; n = 14 cells; p = 0.97; not shown). Application of SR95531 (20 µM, 10 min), a specific GABA_A_ receptor antagonist, blocked IPSPs, abolished the pause, and revealed a stimulation-evoked simple spike firing (Fig. 2A). The frequency of the simple spike firing increased to 65.0±3.5 Hz (asterisk) after the stimulation, which was significantly higher than the basal simple spike firing rate (32.3±1.3 Hz; n = 6 cells; p = 0.005; Fig. 2B). However, SR95531 did not affect the interspike intervals of basal spike firing (artificial cerebrospinal fluid [ACSF]: 29.7±1.0 ms; SR95531: 29.8±0.9 ms; n = 6 cells; p = 0.97; not shown).

**Figure 3:**
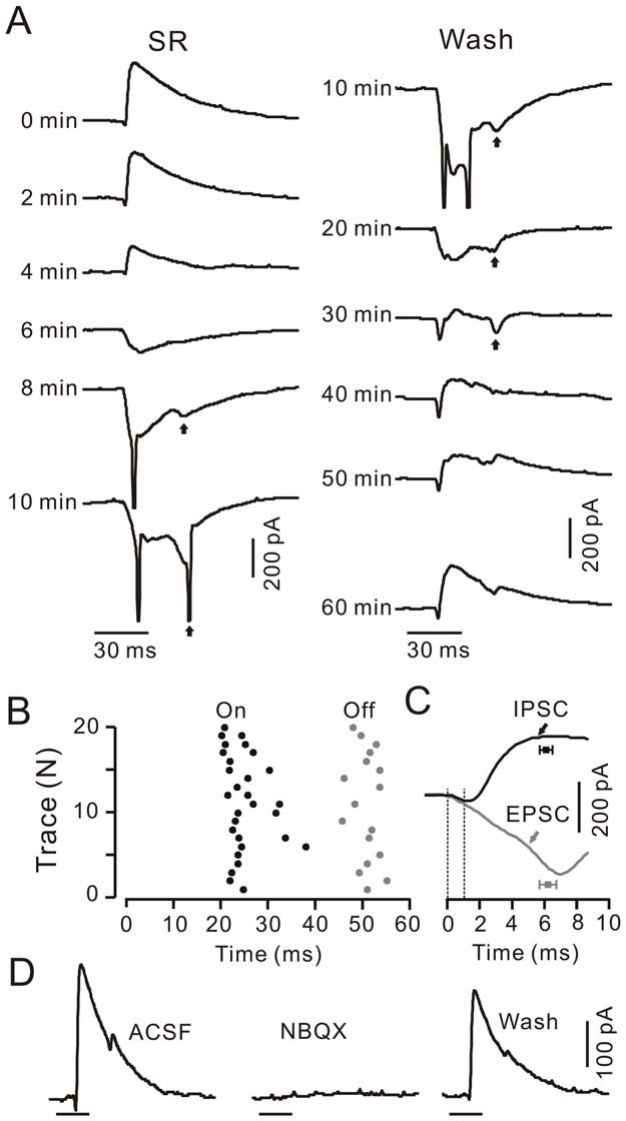
Properties of the somata of PCs in response to tactile stimulation under voltage-clamp. A. Under voltage-clamp (V_hold_  =  −70 mV), the effects of SR95531 (20 µM) on the tactile stimulation (bar, 30 ms)-evoked somatic responses are shown. Arrows indicate the responses evoked by stimulation offset. Note that blockade inhibition revealed the stimulation-evoked action potentials, indicating a poor space clamping of this PC under *in vivo* conditions. B, Raster plot showing the peaks of the responses evoked by onset (On) and offset (Off) of the tactile stimuli (started at t = 0 ms). C, Representative current traces and mean values (± SEM) of the time to peak of the somatic responses evoked by tactile stimuli in ACSF (black; n = 12) and with SR95531 (gray; n = 6). The time point (0, left dashed line) indicates the onset of responses, and the right dashed line indicates the onset of inhibition. D, Under voltage-clamp (V_hold_  =  −70 mV), the somatic response to tactile stimuli (bar, 30 ms) in ACSF, with NBQX (50 µM) and recovery (wash).

Under voltage-clamp conditions (V_hold_  =  −70 mV), application of SR95531 induced a time-dependent decrease in outward currents. Blockade of outward currents revealed an increase in inward currents with a mean amplitude of 80.5±8.6 pA (n = 6 cells). Washout of SR95531 induced a time-dependent decrease in the amplitude of inward currents and a concurrent increase in the amplitude of outward currents (Fig. 3A). These results indicate that the strong inhibition overwhelmed PF-mediated excitatory inputs onto PCs in response to the tactile stimulation. Notably, 90% of the inward currents expressed another peak (Fig. 3A, B) at a mean duration of 28.6±0.5 ms (n = 6 cells) from the first peak, which was of similar delay to the air-puff (30 ms). Thus, both stimulation onset and offset elicit excitatory inputs onto PCs, consistent with the high-fidelity transmission of sensory information by mossy fibers [27]. Importantly, the inhibition was activated within 1.1±0.1 ms (n = 12 cells) after onset of the inward currents in the somata, consistent with the rapid onset of feed-forward inhibition *in vitro* [18]. The inward currents peaked at 6.2±0.5 ms (n = 6 cells), which was similar to the outward currents (6.0±0.4 ms; n = 12 cells; p = 0.86; Fig. 3C). Notably, the 10–90% rise time for inward currents (excitatory postsynaptic currents, EPSCs: 3.7±0.5 ms, n = 6 cells) was significantly slower than that for outward currents (IPSCs: 1.6±0.1 ms, n = 12 cells; p = 0.0002). In addition, NBQX, an AMPA (α-amino-3-hydroxy-5-methyl-4-isoxazole propionate) receptor antagonist reversibly blocked the tactile stimulation-induced tiny inward currents and strong outward currents in the somata of PCs (Fig. 3D; n = 3 cells). Thus, the rapid activation of the feed-forward inhibition overwhelms the excitation of cerebellar somata of PCs in response to air-puff stimulation of the ipsilateral whisker pad.

### Pharmacological properties of the dendrites of PCs in response to tactile stimulation

**Figure 4:**
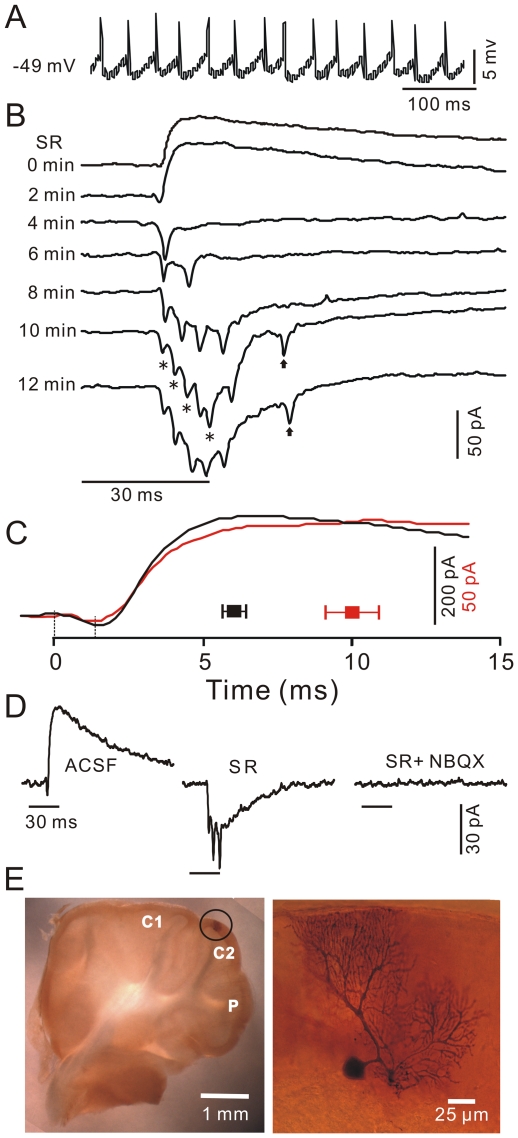
Pharmacological properties of PC dendrites in response to tactile stimulation. A, Under current-clamp (I = 0), a PC dendritic patch-clamp recording demonstrates the spontaneous attenuated simple spike activity. B, Under voltage-clamp (V_hold_  =  −70 mV), the effects of SR95531 (20 µM) on the tactile stimulation (bar, 30 ms)-evoked dendritic responses are shown. Asterisks denote the summation of PF-EPSCs. Arrows indicate the responses induced by stimulation offset. C, Representative current traces and mean values (± SEM) of the time to peak of the responses show the difference between the somata (black; n = 12) and dendrites (red; n = 19) in response to tactile stimulation. The time point (0) indicates the onset of the responses. The vertical scale denotes 200 pA (black) for the soma and 50 pA (red) for the dendrite, respectively. D, Under voltage-clamp (V_hold_  =  −70 mV), the PC dendrites' response to tactile stimuli in ACSF, with SR95531, and with a mixture of SR95531 and NBQX is shown. (E) The photomicrographs depict the morphology of the recorded cell. The left column shows an overview of the location of the biocytin-labeled cell, which is indicated with a black circle. The right column shows the detail of the biocytin-labeled cell.

The dendritic trees of a single cerebellar PC receive about 175,000 excitatory glutamatergic inputs from granule cells [28]. However, tactile stimulation evoked a strong inhibition, not an excitation, in the somata and dendrites of PCs. With the exception of rapid activation of feed-forward inhibition (Fig. 3C), why do PFs express a less powerful response to tactile stimuli? To answer this question, we examined how the dendrites of PCs respond to tactile stimuli. The PC dendritic patch-clamp recording was identified by the attenuated backpropagating of Na^+^ action potentials (Fig. 4A), and confirmed by biocytin histochemistry (Fig. 4E). Under voltage-clamp conditions (V_hold_  =  −70 mV), tactile stimuli evoked a sequence of transient inward currents (6.1±1.3 pA; n = 19 cells) followed by strong outward currents (35.8±6.7 pA; n = 19; Fig. 4B). Application of SR95531 induced a time-dependent decrease in the amplitude of outward currents. Also, blockade of the outward currents revealed a temporal summation of the excitatory inputs of PFs, which were identified as PF-EPSCs (Fig. 4B). The mean frequency of the evoked PF-EPSCs was 266.6±21.1 Hz (n = 7 cells), showing the desynchronization of high-frequency PF activities in response to the tactile stimuli. Thus, these data suggest the asynchronization of PF activity during sensory information processing in the cerebellar cortex. In addition, the stimulation offset-evoked PF-EPSC was also observed in dendrites (Fig. 4B; arrows), and confirmed that the stimulation offset evoked weaker responses than the onset of stimulation. Notably, both the 10–90% rise time (4.0±0.5 ms; n = 19) and time to peak (10.0±0.9 ms; n = 19) of the outward currents in the dendrites were significantly slower than in somata (10–90% rise time: 1.6±0.1 ms; n = 12; p = 0.0001; time to peak: 6.0±0.4 ms; n = 12; p = 0.0004; Fig. 4C). Notably, SR95531 blockade of the outward currents revealed the inward currents with the temporal summation of PF-EPSCs, which were abolished by adding a mix of SR95531 and NBQX (Fig. 4D; n = 3). These findings demonstrate that tactile stimuli induce asynchronous PF excitatory inputs onto dendrites of PCs, which are less powerful than previously believed [7]. Thus, the dendrites of PCs expressed inhibition rather than excitation in response to tactile stimuli.

## Discussion

In this study, we have provided the first patch-clamp recording and pharmacological evidence that the natural stimulation of trigeminal afferents induces synaptic currents in cerebellar PCs *in vivo* in mice. Our results demonstrated that air-puff stimulation on the ipsilateral whisker pad induced asynchronous PF excitatory inputs onto dendrites of PCs. The tactile stimulation did not evoke strong EPSCs and spike firing in PCs, but induced the rapid activation of GABA_A_ receptor-mediated outward currents in the somata and dendrites of PCs in the cerebellar cortex Crus II in urethane-anesthetized mice.

Natural stimulation of trigeminal afferents induced excitation of patch-like excitation of PCs [24], [25]. However, our somatic and dendritic patch-clamp recordings confirmed that tactile stimulation evoked strong IPSCs but not strong EPSCs in the somata and dendrites of PCs. Therefore, the stimulation failed to fire PCs in the cerebellar cortex folium Crus II. Application of the GABA_A_ receptor antagonist SR95531 blocked the IPSPs and revealed simple spike firing in the PCs. Previous work has suggested that sensory stimuli evoked excitation of PCs directly above the activated granule cell patches. The excitation of PCs is dominant within a narrow radius [29]–[31], [24], [25] and is sometimes unresponsive [32]. However, when we examined the distribution of air-puff stimuli evoked-responses in cerebellar cortex folium Crus II, we did not find any excitation patches. The tactile stimulation-induced spike firing of PCs was observed only when GABA_A_ receptor-mediated inhibition was blocked. These contradictory results may be explained as follows. First, the lack of excitatory responses may be due to missing the narrow region of excited PCs because micromapping in mouse cerebellar cortex was not undertaken. A previous micromapping study [2] found that sensory stimuli evoked excitation of PCs directly above the activated granule cell patches in rat cerebellar cortex. However, the mouse cerebellum cortex is smaller than the rat cerebellum, and it would be easy to miss the narrow excitatory region of excited PCs. Secondly, unitary or extracellular recordings have been used frequently in previous studies [2], [14], [29], [30], [33], [34], whereas in this study we employed cell-attached and whole-cell patch-clamp recordings accompanied by pharmacological protocols. Under current-clamp conditions, tactile stimuli evoked strong IPSPs accompanied by a pause in simple spike firing in PCs. Also, SR95531 blocked the IPSPs, abolished the pause and revealed stimulation-evoked action potentials. Somatic and dendritic whole-cell patch-clamp recordings confirmed that tactile stimuli evoked strong IPSCs in the somata and dendrites of PCs. Blockade of inhibition revealed stimulation-evoked EPSCs in the somata and a temporal summation of PF-EPSCs in the dendrites of PCs. Moreover, our results clearly showed that the PCs responded to the onset and offset of tactile stimuli. Long stimulation durations (>50 ms) have been previously reported to elicit ON and OFF excitatory components in response to the stimulation onset and offset in the cerebellum, respectively [19]. Our present study showed that the offset response was elicited by an air-puff stimulation of 30 ms duration, which is consistent with the high-fidelity transmission of sensory information by mossy fibers [27]. These results suggest that low threshold vibration-sensitive mechanoreceptors generated the receptor potentials at both stimulation onset and offset. However, the long-lasting excitation of PCs induced by electrical stimulation of trigeminal afferents but not by air-puff stimulation of the whisker pad [33] indicates that electrical stimulation of the whisker pad induced non-selective activation of distinct mechanoreceptors. In addition, Morissette and Bower [34] have shown two distinct stimulation onset response peaks in the cerebellar granule cell layer. The second response peak has a cortical origin and a latency of approximately 20–25 ms. However, our results showed that the second response evoked by stimulation offset has a cerebellar origin. The duration between the two responses was not fixed at approximately 30 ms, and corresponded to the various durations of air-puff stimulation. On the other hand, the tactile stimulation-induced PF excitatory inputs onto PC dendrites are less powerful than previously believed [35], because they are asynchronous. These asynchronous inputs require several (∼6) milliseconds of PF excitatory inputs to provoke spike firing in PCs, which is similar to the findings of an *in vitro* study [36]. However, the activation of molecular interneurons is rapid [23] and is synchronized through gap junctions [23], [36]. Consequently, tactile stimuli merely evoked tiny EPSCs (Fig. 2C) that were insufficient to evoke an action potential in the somata of PCs [37]. Taken together, natural stimuli of trigeminal afferents evoked strong inhibition rather than simple spike firing of PCs in the cerebellar cortex Crus II of urethane-anesthetized mice.

Both the anatomical and physiological features of cerebellar circuits are consistent with our results. The PCs receive at least two sets of subcellularly targeted inhibitory inputs. These two inhibitory inputs are the stellate cell axons, which selectively innervate PC dendrites, and the basket cells, which project axon terminals to the axon initial segment to form pinceau synapses and initiate perisomatic inhibition of PCs [5], [18], [24]. The molecular layer interneurons are small with high-input resistance, express a low threshold for activation [25], and can reliably trigger spike outputs after stimulation of a single PF [37]. The molecular layer interneurons are electrically coupled, and an input to one interneuron can activate a group of interneurons through gap junctions [36]. In addition, the tactile stimulation-induced PF excitatory inputs onto PC dendrites are asynchronous and less powerful than previously believed, and a temporal summation of the PF excitatory inputs is required for firing the PCs. The inhibition of PCs together with the asynchronous activities of PFs provide strong evidence to explain how natural activation of PFs has failed to activate the beams of PCs, and provide new functional insights into compartmentalization of the cerebellar hemisphere cortex.

## Materials and Methods

### Anesthesia and surgical procedures

The anesthesia and surgical procedures have been described previously [26]. In brief, experimental procedures were approved by the Animal Care and Use Committee of Jilin University and were in accordance with the animal welfare guidelines of the National Institutes of Health. The permit number was SYXK(Ji)2007-0011. Forty-eight adult (6–8-week-old) HA/ICR mice were anesthetized with urethane (1.3 g/kg body weight i.p.). The mice were tracheotomized to avoid respiratory obstruction and fixed on a custom-made stereotaxic frame. After a watertight chamber was created, a 1–1.5 mm craniotomy was drilled to expose the cerebellar surface corresponding to Crus II. The brain surface was constantly superfused with oxygenated ACSF (composition in mM: 125 NaCl, 3 KCl, 1 MgSO_4_, 2 CaCl_2_, 1 NaH_2_PO_4_, 25 NaHCO_3_, and 10 D-glucose) with a peristaltic pump (Gilson Minipulse 3; Villiers, Le Bel, France) at 0.4 ml/min. Rectal temperature was monitored and maintained at 37.0±0.2°C by body temperature equipment.

### Stimulation and drug application

Tactile stimulation on the ipsilateral whisker pad was performed by air-puff (30–90 ms, 50 psi) through a 12-gauge stainless steel tube connected with a pressurized injection system (Picospritzer® III; Parker Hannifin Co., Pine Brook, NJ). The air-puff was delivered at 0.33 Hz. All the drugs were dissolved in ACSF, and applied onto the cerebellar surface at 0.4 ml/min. SR95531 hydrobromide (6-imino-3-(4-methoxyphenyl)-1 (6H)-pyridazinebutanoic acid hydrobromide) and NBQX (2,3-dioxo-6-nitro-1,2,3,4- tetrahydrobenzo[f] quinoxaline-7- sulfonamide) were purchased from Tocris Cookson (Bristol, UK). Tetrodotoxin was purchased from Sigma (Sigma-Aldrich, Shanghai, China).

### Electrophysiological recording and biocytin histochemistry

The somatic and dendritic patch-clamp recordings from PCs were performed with an Axopatch-1D amplifier (Molecular Devices, Foster City, CA). The synaptic potentials (currents) were acquired through a Digidata 1200 series analog-to-digital interface on a personal computer using Clampex 8.1 software. Patch electrodes (4–6 MΩ) contained a solution of the following composition (in mM): potassium gluconate 120, HEPES 10, EGTA 1, KCl 5, MgCl_2_ 3.5, NaCl 4, biocytin 8, Na_2_ATP 4, and Na_2_GTP 0.2 (pH 7.3 with KOH, osmolarity adjusted to 300 mOsm). After the experiments, the whole brain was removed and fixed in 4% paraformaldehyde in 0.1 PBS (pH 7.4) at 4°C for 24 hours. Slices were cut in the sagittal plane at 200 µm using a vibratome (XY-86; Zhejiang, China), and washed with PBS. The tissue was reacted overnight with an avidin-biotin complex (ABC Elite kit; Vector Laboratories, Burlingame, CA) at 4°C. Finally, biocytin binding was visualized by 3,3′-diaminobenzidine tetrahydrochloride histochemistry.

### Statistical analysis

Electrophysiological data were analyzed using Clampfit 8.1 software (Molecular Devices, Foster City, CA). Values were expressed as the mean ± SEM. Differences between the mean values recorded under control and test conditions were evaluated by Student's paired *t*-test or one-way ANOVA using SPSS (Chicago, IL) software.
